# Intakes of Folate and Vitamin B12 and Biomarkers of Status in the Very Old: The Newcastle 85+ Study

**DOI:** 10.3390/nu8100604

**Published:** 2016-09-28

**Authors:** Nuno Mendonça, John C. Mathers, Ashley J. Adamson, Carmen Martin-Ruiz, Chris J. Seal, Carol Jagger, Tom R. Hill

**Affiliations:** 1 School of Agriculture Food and Rural Development, Newcastle University, Newcastle upon Tyne NE1 7RU, UK; Chris.seal@newcastle.ac.uk (C.J.S.); Tom.hill@newcastle.ac.uk (T.R.H.); 2 Newcastle University Institute for Ageing, Newcastle University, Newcastle upon Tyne NE2 4AX, UK; john.mathers@newcastle.ac.uk (J.C.M.); Ashley.Adamson@newcastle.ac.uk (A.J.A.); Carmen.martin-ruiz@newcastle.ac.uk (C.M.-R.); carol.jagger@newcastle.ac.uk (C.J.); 3 Human Nutrition Research Centre, Newcastle University, Newcastle upon Tyne NE2 4HH, UK; 4 Institute of Cellular Medicine, Newcastle upon Tyne NE2 4HH, UK; 5 Institute of Health and Society, Newcastle University, Newcastle upon Tyne NE2 5PL, UK

**Keywords:** ‘aged 80 and over’, Newcastle 85+ Study, red blood cell folate, vitamin B12, *FUT2*, *MTHFR*, food groups

## Abstract

Very old adults are at increased risk of folate and vitamin B12 deficiencies due to reduced food intake and gastrointestinal absorption. The main aim was to determine the association between folate and vitamin B12 intake from total diets and food groups, and status. Folate or vitamin B12 intakes (2 × 24 h multiple pass recalls) and red blood cell (RBC) folate or plasma vitamin B12 (chemiluminescence immunoassays) concentrations were available at baseline for 731 participants aged 85 from the Newcastle 85+ Study (North-East England). Generalized additive and binary logistic models estimated the associations between folate and vitamin B12 intakes from total diets and food groups, and RBC folate and plasma B12. Folate intake from total diets and cereal and cereal products was strongly associated with RBC folate (*p* < 0.001). Total vitamin B12 intake was weakly associated with plasma vitamin B12 (*p* = 0.054) but those with higher intakes from total diets or meat and meat products were less likely to have deficient status. Women homozygous for the *FUT2* G allele had higher concentrations of plasma vitamin B12. Cereals and cereal products are a very important source of folate in the very old. Higher intakes of folate and vitamin B12 lower the risk of “inadequate” status.

## 1. Introduction

B vitamins, specifically folate and vitamin B12, are essential for one-carbon transfer reactions which include amino acid interconversions, RNA and DNA synthesis and methylation of cell macromolecules [1]. Older adults are at increased risk of B vitamin deficiencies due to decreased food intake and increased malabsorption. Low folate and vitamin B12 status have been associated with adverse health outcomes, including cognitive impairment [2,3,4], stroke [5,6], fractures [7,8] and cancer in older adults [9]. A review of micronutrient deficiencies in community-dwelling older adults (aged 65 and over) living in western countries reported that 29% and 16% of men and, 30% and 19% of women had intakes below the Nordic Nutrition Recommendations (NNR) estimated average requirement (EAR) for folate (200 µg/day) and vitamin B12 (1.4 µg/day), respectively [10]. The current UK National Diet and Nutrition Survey (NDNS) rolling programme estimated that 1% of older adults (aged 65 and over) were below the UK lower reference nutrient intake (LRNI) for folate (100 µg/day) and vitamin B12 (1.0 µg/day) but that 7.3% of men and 10.8% of women had red blood cell folate (RBC) concentrations below 340 nmol/L and 5.9% of men and women had serum vitamin B12 concentrations below 150 pmol/L [11]. The complexity of the dose–response relationships between intake and status is influenced by limitations in dietary assessment, food composition data, choice of biomarkers, genotypic variation, bioavailability and complex metabolic pathways. About 10%–30% of older adults have atrophic gastritis (caused by *Helicobacter pylori* infection, long-term use of proton pump inhibitors, H_2_ receptor antagonists and biguanides) which leads to hypochlorhydria [12]. This has a detrimental effect onacid–pepsin digestion and favours small bowel bacterial growth resulting in impaired vitamin B12 absorption [13]. In addition, those with autoimmune atrophic gastritis produce antibodies against the intrinsic factor which can lead to pernicious anemia [13]. Therefore, older adults may have adequate vitamin B12 intake but inadequate vitamin B12 plasma concentration. In addition, several single nucleotide polymorphisms (SNP) modulate folate and vitamin B12 status. For example, homozygosity of the T allele (forward orientation) (rs1801133) of the *MTHFR* gene (which encodes methylenetetrahydrofolate reductase) is associated with low folate status [14].

There is conflicting evidence about relationships between folate and vitamin B12 intake and, folate and vitamin B12 status, respectively, in older adults. Some studies report a significant association between folate and vitamin B12 intake and status in older adults [2,15,16,17,18,19] while others do not [20,21,22]. Differences in folate and vitamin B12 bioavailability from total diets and specific food sources may provide a partial explanation for the observed discrepancies. Folate bioavailability from foods is substantially lower than that from supplements or from foods fortified with folic acid with estimated bioavailability of 50% and 85%, respectively [23]. If intrinsic factor (IF) secretion is intact, approximately 40% of vitamin B12 is absorbed [24].

In light of the concerns about dietary inadequacy, it is imperative to assess folate and vitamin B12 status in older people, particularly the very old (85 years and older). The aims were to determine (i) the prevalence of “inadequate” folate and vitamin B12 intake and status in the Newcastle 85+ Study; (ii) the associations between the top contributing dietary sources of folate and vitamin B12, and status; and (iii) whether high dietary intakes of both vitamins are associated with a reduced risk of “inadequate” status.

## 2. Material and Methods

### 2.1. Newcastle 85+ Study

The Newcastle 85+ Study is a longitudinal population-based study of health trajectories and outcomes in the very old which approached all people turning 85 in 2006 (born in 1921) who were registered with participating general practices within Newcastle upon Tyne or North Tyneside primary care trusts (North East England). Details of the study have been reported elsewhere [25,26,27]. All procedures involving human subjects were approved by the Newcastle and North Tyneside local research ethics committee (06/Q0905/2). Written informed consent was obtained from all participants, and when unable to do so, consent was obtained from a carer or a relative. The recruited cohort was socio-demographically representative of the general UK population [25]. At baseline (2006/2007), multidimensional health assessment, complete general practice (GP) medical records data and complete dietary intake data (without protocol violation) were available for 793 participants [28].

### 2.2. Dietary Assessment and Food Groups

Dietary intake was collected at baseline using two 24 h Multiple Pass Recalls (24 h-MPR) on two non-consecutive occasions in the participant’s usual residence by a trained research nurse and energy, folate and vitamin B12 intakes were estimated using the McCance and Widdowson's Food Composition tables 6th edition [29]. Individual foods were coded and allocated to 15 first level food groups that consisted of: cereals and cereal products, milk and milk products, eggs and egg dishes, oils and fat spreads, meat and meat products, fish and fish dishes, vegetables, potatoes, savoury snacks, nuts and seeds, fruit, sugar, preserves and confectionery, non-alcoholic beverages, alcoholic beverages and miscellaneous (soups, sauces and remaining foods that did not belong in other food groups) [28]. The top three food group contributors to folate or vitamin B12 intakes (accounted for >50% of total intake) were included in the analysis. These food groups were also widely consumed by this population and, therefore, a possible target for public health policies/fortification. Information on supplement use was limited to type and brand and, therefore, this was only used as a dichotomous covariate (yes/no) [30].

### 2.3. Nutritional Biomarkers and Single Nucleotide Polymorphisms

Blood samples were taken after an overnight fast at baseline. Forty mL of blood was drawn from the antecubital vein between 7:00 a.m. and 10:30 a.m., placed on ethylenediamine tetraacetic acid (EDTA) tubes and 95% of the samples were taken to the laboratory within 1 h [31]. Red blood cell folate (RBC folate) and plasma vitamin B12 concentrations were determined by chemiluminescence (Microparticle Immunoassay on an Abbott ARCHITECT analyser) and data were available for 731 and 732 participants, respectively. RBC folate was stabilized with ascorbic acid and adjusted for hematocrit. Whole blood DNA was extracted by means of a QiaGEN Amp Maxi DNA Purification Kit. As part of the EU Longevity Genetics Consortium, genome-wide association studies (GWAS) were performed on 765 participants from the Newcastle 85+ Study using Illumina Omni genotyping arrays. Data were obtained from 710 individuals and after quality control, 642 individuals were retained for the final analysis [32]. The single nucleotide polymorphisms (SNP) in the *MTHFR* (rs1801133, chromosome 1, position 11796321), *FUT2* (rs492602, chromosome 19, position 48703160), *MTR* (rs1805087, chromosome 1, position 236885200) and *TCN1* (rs526934, chromosome 11, position 59866020) genes were chosen as candidate modifiers of RBC folate and plasma vitamin B12 concentrations. All SNPs were assessed for deviation from the Hardy–Weinberg equilibrium.

### 2.4. Statistical Analysis

Normality was assessed graphically with the aid of Q-Q plots and histograms. Linearity and homoscedasticity assumptions were tested with residuals versus predicted values plots. Normally distributed continuous values are presented as means and standard deviations (SD), and non-Gaussian distributed variables as medians and interquartile ranges (IQR). Categorical data are presented as percentages (with corresponding sample size). Sex differences were assessed with the Chi-squared test (χ^2^) for categorical variables and by independent *t*-test, and Mann–Whitney U test for parametric and non-parametric continuous data, respectively. Differences between RBC folate and plasma vitamin B12 concentrations according to *MTHFR* (rs1801133), *MTR* (rs1805087), *TCN1* (rs526934) and *FUT2* (rs492602) genotype were assessed using Kruskal–Wallis tests followed by Dunn–Bonferoni tests if the null hypothesis was rejected.

The semi-parametric generalized additive models (gam) were investigated in R version 3.0.1 (R foundation for statistical computing) using the package “gam” to plot the relationship between vitamin intakes (thin plate regression splines for smoothing) and corresponding biomarkers. The generated reference value of zero in the y-axis corresponds to the RBC folate/ plasma vitamin B12 concentrations for the mean intake of folate and vitamin B12, respectively. The odds ratio (OR) (and 95% confidence intervals (CI)) of RBC folate concentrations <600 nmol/L and plasma B12 <148 pmol/L according to quartiles of folate and vitamin B12 intake from total diets and top contributing food groups were computed using binary logistic regression. The commonly used cut-off to define folate deficiency of RBC folate <340 nmol/L [23] could not be used for these models due to the low percentage of deficiency among study participants. Gam and binary logistic regression models were adjusted for sex, energy intake, folic acid or vitamin B12 containing supplement use, folate/vitamin B12 intake from other food groups, *MTHFR* or *FUT2* genotype. The vitamin B12 models were additionally adjusted for H_2_ receptor antagonists, biguanides and proton pump inhibitor (PPI) use.

*p* < 0.05 was considered statistically significant. Apart from the gam models, all statistical analyses were conducted using the IBM statistical tool SPSS v22 (IBM, New York, NY, USA).

## 3. Results

### 3.1. Folate and Vitamin B12 Intake and Status “Inadequacies”

Although 43% of participants (*n* = 335) consumed one or more supplements on a regular basis [30], only 4.8% were users of folic acid and vitamin B12 as part of multivitamin supplements (Table 1). A low percentage of participants (3.1%) had folate intakes below the UK LRNI (100 µg/day) [33] or had RBC folate concentrations below the classic cut-off for deficiency of 340 nmol/L (3.6%) [23]. Folate intake and status were “inadequate” in only five participants. Cereals and cereal products, vegetables and fruit and fruit juice were the top food group contributors to folate intake, explaining almost 60% of total folate intake. Vitamin B12 intakes were below the UK LRNI (1 µg/day) [33] in 9.2% (*n* = 67) of the population while 17.1% (*n* = 125) were below 148 pmol/L of plasma vitamin B12 [24] (110 of these 125 had also total homocysteine concentrations >15 µmol/L). In addition, 17 participants had “inadequate” intakes and also deficient plasma concentrations of vitamin B12. There were twice as many women with vitamin B12 intakes below the UK LRNI than men (5.0% vs. 12.4%, *p* < 0.001) but not based on plasma vitamin B12 concentrations <148 pmol/L (17.4% vs. 16.9%, *p* = 0.238). Eighty-six percent (*n* = 628) of the participants had plasma vitamin B12 concentration <400 pmol/L, a concentration that has been associated with high total homocysteine and methylmalonic acid concentrations [34,35]. Intake of the top three food groups (meat, fish and dairy) explained more than 80% of total vitamin B12 intake.

### 3.2. Folate, Vitamin B12 Status and Genotype

RBC folate and plasma vitamin B12 concentrations according to *FUT2, MTHFR, MTR* and *TCN1* and genotypes are shown in Table 2. Individuals with the *MTHFR* (rs1801133) AG or GA genotype [minor allele frequency (MAF) = 0.33 in the Newcastle 85+ Study vs. 0.32 for the A allele from the 1000 Genomes Project British population phase 3 [36]] had higher RBC folate concentrations than those homozygous for G (*p* = 0.024). Participants with the *FUT2* (rs492602) GG genotype had higher concentrations of plasma vitamin B12 than other *FUT2* genotypes (*p* < 0.001) (MAF = 0.45 in the Newcastle 85+ Study vs. 0.48 for the G allele in residents of England and Scotland [36]). The association between *FUT2* genotype and plasma vitamin B12 concentrations was significant in women (*p* < 0.001) but not in men (*p* = 0.140).

### 3.3. Association between Folate Intake and Status

**Figure 1:**
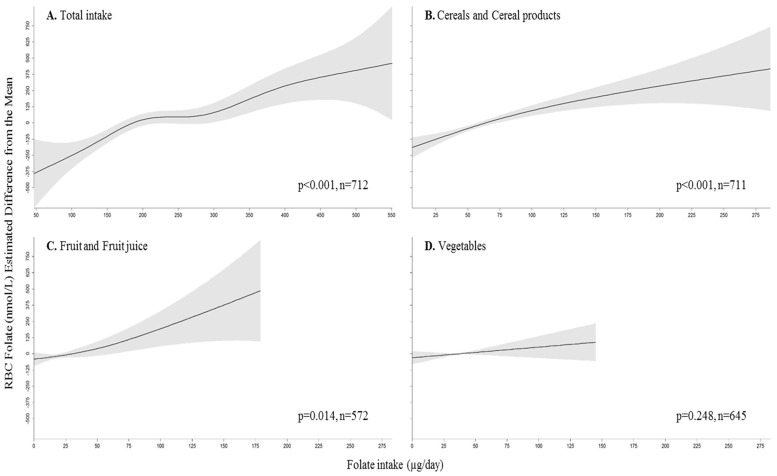
Estimated difference from the mean (and 95% CI) of RBC folate concentration according to folate intake from A. all dietary sources, B. from cereals and cereal products, C. from fruit and fruit juice and D. from vegetables. Generalized additive model (gam) adjusted for sex, energy intake, *MTHFR* genotype, folic acid supplement use and folate intake from the two other food sources. The highest 2.5th percentiles of RBC folate concentrations are not included. Three participants with a folate intake above 150 µg only from vegetables were not included. One participant with a folate intake of 327 µg only from fruit and fruit juice was excluded. *p* values are from the corresponding gam model.

The associations between folate intake from all food sources, from cereals and cereal products, from fruit and fruit juice and from vegetables, and RBC folate concentrations are shown in Figure 1 (gam model adjusted for sex, energy intake, *MTHFR* genotype, folate intake from the two other food groups and folic acid supplement use). Total folate intakes were associated with RBC folate (*p* < 0.001). The steepest part of the dose–response curve appeared to be for folate intakes of 50–200 µg per day but RBC folate concentrations continued to increase with increasing folate intake up to ≈500 µg per day. Folate intake from cereals and cereal products, and from fruit and fruit juice were also associated with RBC folate concentrations (*p* < 0.001 and *p* = 0.014, respectively) (Figure 1).

### 3.4. Risk of Low Folate Status by Folate Intake

Table 3 shows the odds ratio (OR) (and 95% CI) of low RBC folate (<600 nmol/L) according to total, cereals and cereal products, vegetables and, fruit and fruit juice folate intake quartiles. Individuals in the highest quartile of total folate intake (>264 µg/day) were less likely to have RBC folate concentrations <600 nmol/L than those in the lowest quartile (<157 µg/day) in the unadjusted model (OR: 0.58, 95% CI: 0.36, 0.94) and adjusted model (OR: 0.43, 95% CI: 0.23, 0.82). Individuals in higher quartiles of folate intake from cereals and cereal products and from vegetables were also less likely to have low RBC folate concentrations (<600 nmol/L) than those in quartile 1. The same was not true for folate intake from fruit and fruit juice in any model.

### 3.5. Association between Vitamin B12 Intake and Status

**Figure 2:**
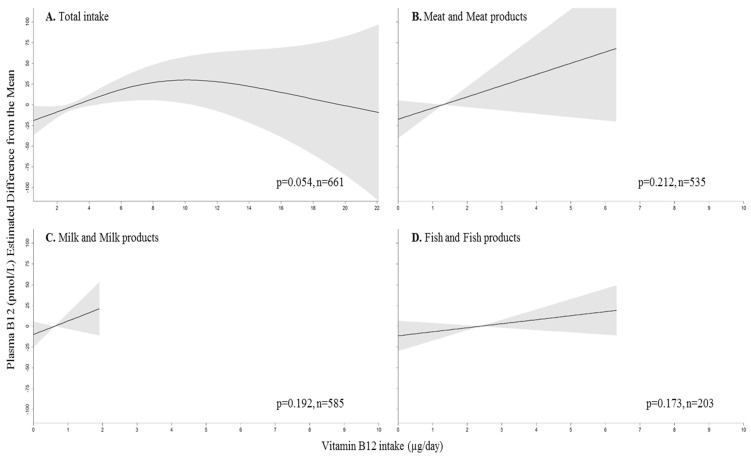
Estimated difference from the mean (and 95% CI) of plasma B12 concentrations according to vitamin B12 intake from A. all dietary sources, B. from meat and meat products, C. from milk and milk products and D. from fish and fish products. Generalized additive model (gam) adjusted for sex, energy intake, *FUT2* genotype, H_2_ antagonists, proton pump inhibitors or biguanides use, vitamin B12 supplement use and vitamin B12 intakes from the other two food sources. The lowest and highest 2.5th percentiles of vitamin B12 intakes and plasma vitamin B12 concentrations are not included except for meat and meat products where the highest 5th percentile was excluded. *p* values are from the corresponding gam model.

Total vitamin B12 intake was weakly associated with plasma vitamin B12 concentrations while adjusting for sex, energy intake, vitamin B12 intake from the other two food groups, *FUT2* genotype, vitamin B12 supplement use and H_2_ antagonists, biguanides or PPI use (*p* = 0.054) (Figure 2). Plasma vitamin B12 concentration appeared to decrease when vitamin B12 intake exceeded 10 µg/day but the CI were very wide thereafter. Vitamin B12 intake from meat and meat products, milk and milk products and fish and fish dishes were not associated with plasma vitamin B12 concentration.

### 3.6. Risk of Deficient Vitamin B12 Status by Vitamin B12 Intake

Participants with total vitamin B12 intake above the median (2.88 µg/day) were half as likely to be deficient for plasma B12 as those with the lowest intake (<1.87 µg/day) in the adjusted model (Table 4). Individuals in quartile 4 of vitamin B12 intake from meat and meat products (>2.10 µg/day) were also half as likely to be deficient for plasma vitamin B12 as those in quartile 1 in the unadjusted (OR: 0.55, 95% CI: 0.31–0.98) and adjusted models (OR: 0.41, 95% CI: 0.20–0.81). The same trend was present for milk and milk products but this did not reach statistical significance (*p* = 0.054).

## 4. Discussion

This study found that, in the very old, folate intakes from all food sources and from cereals and cereal products were significantly associated with RBC folate. Individuals with higher total folate intake or intake from cereals and cereal products were less likely to have low concentrations of RBC folate. The association between vitamin B12 intakes and plasma vitamin B12 was weak. Individuals with vitamin B12 intakes from all food sources and from meat and meat products were also less likely to be deficient for plasma vitamin B12. In addition, higher concentrations of RBC folate were found in participants with the *MTHFR* (rs1801133) AA genotype compared with those with A/G or GG genotypes. Women homozygous for the *FUT2* (rs492602) G allele also had higher concentrations of plasma vitamin B12 than those with other *FUT2* genotypes.

### 4.1. Folate and Vitamin B12 Intake and Status “Inadequacies”

In the Newcastle 85+ Study there was a relatively low prevalence of “inadequate” folate intake (3.1%) and status (3.6%). The NDNS rolling programme estimated that 1% of older adults (aged 65 and over) were below the UK LRNI for folate but that 7.3% of men and 10.8% of women had RBC folate concentrations <340 nmol/L. In the Newcastle 85+ Study, plasma vitamin B12 deficiency (<148 pmol/L) was present in 17.1% of participants and 9.2% were below the UK LRNI (1 µg/day) for vitamin B12 intake whilst the NDNS estimated that 1% were below the LRNI for vitamin B12 but 5.9% had serum vitamin B12 concentrations <150 pmol/L [11]. Age, dietary assessment method (4-day weighted diet record vs. 2 × 24 h-MPR) and other methodological differences are likely explanations for these observed discrepancies. Specifically, the novel method used to assess RBC folate in the NDNS (whole blood folate by a microbiological assay, serum total folate by LC-MS/MS and hematocrit) is likely to give higher estimates of folate “inadequacy” than the one used in this study. The NDNS used a similar method to the Newcastle 85+ Study to assess plasma vitamin B12 (competitive immunoassay with direct chemiluminescence (ADVIA Centaur B12 assay)).

In the post-fortification period in the US, less than 1% of older adults had deficient folate status (RBC folate <340 nmol/L) [37]. In the National Health and Nutrition Examination Survey (NHANES) 2003–2006, it was estimated that 9% of men and 24% of women >70 years old were below the North American EAR for folate (320 DFE/day [38,39]). In the same NHANES edition, 19% of older adults had plasma vitamin B12 concentrations below 221 pmol/L [40]. Moreover, less than 1% of men and 6% of women >70 years were below the EAR for vitamin B12 (2 µg/day) [39]. Almost 30% (*n* = 235) of the Newcastle 85+ Study participants were below the same EAR for vitamin B12 intake.

### 4.2. Association between Folate Intake and Status

Folate intake from total diets and from cereals and cereal products but not from vegetables or fruit and fruit juice were associated with RBC folate concentrations in the very old. Vegetables and fruit and fruit juice contributed to 16% and 9%, respectively to folate intake and the relatively lower contribution might explain the lack of associations. Further, folate bioavailability is dependent on the food matrix, stability of labile folates, presence of vitamin C and folate-binding proteins and folate pool sizes [23,41,42]. Nonetheless, there is a consensus that folic acid is better absorbed than dietary folate. Evidence also shows that that folic acid intake is a stronger predictor of RBC folate concentration than total folate intake [43]. The US Institute of Medicine estimated that the absorption efficiency of folic acid in supplements or fortified food was 85% taken with food or 100% from supplements taken on an empty stomach [23], whilst dietary folate absorption efficiency was 50% [23,42]. Breakfast cereals (grouped under cereals and cereal products in this study) have frequently been the target of voluntary fortification in the UK and elsewhere which might explain the stronger association between folate intake from cereals and cereal products and RBC folate concentrations. Cereals and cereal products were also the top contributors to folate intake (32%), suggesting that this is an important source of folate/folic acid in this population group. On the other hand, the incomplete release of dietary folate from plant foods cellular structures may explain a weaker association between folate from vegetables and fruit, and RBC folate.

### 4.3. Association between Vitamin B12 Intake and Status

Total vitamin B12 was weakly associated with plasma B12 in the very old (*p* = 0.054) and seemed to saturate at intakes ≈10 µg. The relatively weak association might be due to the low vitamin B12 intakes in relation to the large liver stores (1 µg/g of liver) so that intakes only slowly influence plasma concentrations [24]. Further, vitamin B12 absorption is complex. Bound to protein in food, vitamin B12 has to be released by pepsin and hydrochloric acid in the stomach. The ensuing free form of vitamin B12 binds to haptocorrin, forming a B12-haptocorrin complex. This complex is later broken down in the small intestine by pancreatic proteases which enable vitamin B12 to bind to the glycoprotein IF, be recognized (by cubilin) and absorbed by endocytosis in the enterocytes of the distal ileum. These steps present a problem to older adults as 10%–30% have atrophic gastritis and therefore, reduced gastric acid secretion which is essential to vitamin B12 release from food proteins [12]. The bioavailability of vitamin B12 in any form or dose is estimated to be 40% in healthy adults with intact IF secretion [24]. Meat and meat products, milk and milk products and, fish and fish products were the top sources of vitamin B12 in the Newcastle 85+ Study [30]. However, unlike some findings [44,45], but in agreement with others [46], vitamin B12 intake from meat and meat products was not associated significantly with plasma vitamin B12. Meat and meat products, especially liver (beef liver can reach to as much as 83 µg per 100 g [29]) and ruminant meat have very high concentrations of vitamin B12 and it is reported that ileal receptors saturate with intakes of 1.5–2.5 µg of vitamin B12 per meal [47]. Only 50% and 5% of vitamin B12 are absorbed with intakes of ~1 and 25 µg, respectively [24]. Others have found that vitamin B12 from dairy products is very bioavailable [48] but also that vitamin B12 in yogurt and cheese is not as bioavailable as that in milk in older individuals [46]. This could explain why vitamin B12 intake from dairy products (that includes yogurt and cheese) was not associated with plasma B12 in this study.

### 4.4. MTHFR and RBC Folate, and FUT2 and Plasma Vitamin B12

Interestingly, and in contrast to most previous findings [49], participants heterozygous for the A allele of the *MTHFR* gene had higher concentrations of RBC folate than those homozygous for the G allele. This was not a reflection of higher folate or folic acid containing supplements intake. Similar to previous findings [14,49,50], women with the *FUT2* GG genotype had higher concentrations of plasma vitamin B12. *FUT2* encodes galactoside 2-alpha-*L*-fucosyltransferase 2 (EC: 2.4.1.69) which is involved in the regulation of the H antigen and is a precursor of the ABO (H) antigens [50]. *FUT2* variants (from the allele A) are proposed to be protective against *Helicobacter pylori* infection or to increase IF production [14]. Both proposed explanations would explain the higher plasma vitamin B12 concentrations in those homozygous for the G allele.

### 4.5. Strengths and Weaknesses

The Newcastle 85+ Study is a unique cohort owing to the age group, the large number of participants and the extensive multidimensional health data. The study was socio-demographically representative of the UK but the lack of ethnic diversity warrants caution when generalizing the findings to a non-white population. The rapid processing of blood samples after venepuncture is another strength of this study.

As dietary intake assessment consisted of a 24 h-MPR applied on two non-consecutive days, the possibility of unusually high or low vitamin B12/folate intakes cannot be excluded. For practical reasons, 24 h-MPRs were not conducted during the weekend, therefore food and drink eaten on Fridays and Saturdays was not recalled. Although the MPR method involved several prompts to avoid misreporting, misreporters have been estimated to be 26% of the cohort [28]. Even though the food groups used in the analysis contributed to most of the folate and vitamin B12 intake, other food sources might explain the remaining intake. Intakes of dietary folate equivalents (DFE) and of the crystalline form of vitamin B12 could not be determined because supplement use was collected qualitatively (type and brand but not frequency) and it was not certain which specific foods had been fortified during the dietary collection period (2006/2007). A general limitation of most dietary surveys, including ours, is that assessment of supplement usage may not be accurate by dietary intake records, dietary recalls and other questionnaires [51]. Furthermore, the irregular use of supplements by survey participants, including the alteration of usual patterns of supplement use during the period of dietary data collection, further adds bias to the estimation of true supplement use [51]. It is worth mentioning that the choice of vitamin B12 form used in supplements and fortified foods should also be taken into consideration because of concerns associated with cyanide/thiocyanate from cyanocobalamin [52].

Holotranscobalamin measures the vitamin’s active form and because it might better reflect vitamin B12 status than plasma vitamin B12, its use might have yielded different results. There is currently no consensus on the biochemical threshold to use in order to define folate or vitamin B12 “inadequacy”, especially in this population. Therefore, results from the binary logistic models might be different if different thresholds were used. Furthermore, atrophic gastritis impairs folate and vitamin B12 absorption. If available, the incidence of atrophic gastritis or a proxy measure, such as *Helicobacter pylori* infection, could have been used as an adjusting factor or to conduct a sensitivity analysis. The list of SNPs used is not exhaustive and other polymorphisms, such as some SNPs in the *TCN2* gene (e.g., rs731991) may influence the folate and vitamin B12 intake–status relationship [53].

## 5. Conclusions

In summary, almost one-fifth of the 85-year-old participants in the Newcastle 85+ Study had deficient plasma vitamin B12 concentrations but only a few individuals had deficient folate status, according to commonly used biochemical thresholds. Folate and vitamin B12 intakes were associated to RBC folate and plasma B12, respectively and folate intake from cereals and cereal products was strongly associated with RBC folate. This is possibly a consequence of voluntary folic acid fortification of breakfast cereals in the UK, and makes this food group an important source of folate for this population group. Estimates of the bioavailability of folate and vitamin B12 from total diets and from commonly consumed food groups in the very old should be taken into account when setting dietary guidelines.

## Figures and Tables

**Table 1:** Population characteristics, folate and vitamin B12 intakes and biomarkers of one carbon metabolism in the Newcastle 85+ Study.

	All	Men	Women	*p*-Value ^1^
Sex (%) (*n*)	732	39 (287)	61 (445)	-
BMI (kg/m^2^) (mean ± SD)	24.4 ± 4.3	24.7 ± 3.9	24.3 ± 4.6	0.244 ^2^
Smokers (%) (*n*)	5.6 (41)	4.2 (12)	6.5 (29)	0.183
Alcohol Drinkers (%) (*n*)	72 (364)	84 (192)	62 (172)	<0.001
Total Energy Intakes (MJ/day)	6.78 (5.62–8.31)	8.01 (6.65–9.59)	6.26 (5.17–7.38)	<0.001
Folate and vitamin B12 supplement use (%) (*n*)	4.8 (35)	3.8 (11)	5.4 (24)	0.334
H_2_ antagonists, PPI and biguanides use (%) (*n*)	26.8 (196)	27.2 (78)	26.5 (118)	0.844
Total Homocysteine (µmol/L)	16.7 (13.5–21.4)	18.0 (14.5–21.9)	16.1 (13.1–21.0)	0.001
>15 µmol/L (%) (*n*)	63.1 (471)	70.3 (206)	58.5 (265)	0.001
**Folate**				
Intake (µg/day)	209 (157–265)	246 (185–296)	189 (144–242)	<0.001
<100 µg/day (%) (*n*)	3.1 (23)	0.7 (2)	4.7 (21)	0.002
Top food group contributors	Cereals (32%), Vegetables (16%), Fruit (9%)	Cereals (32%), Vegetables (15%), Fruit (8%)	Cereals (31%), Vegetables (17%), Fruit (10%)	-
Red Blood Cell Folate (nmol/L)	863 (451–1287)	868 (596–1282)	854 (614–1287)	0.728
<340 nmol/L (%) (*n*)	3.6 (26)	2.1 (6)	4.5 (20)	0.103
**Vitamin B12**				
Intake (µg/day)	2.9 (1.9–4.4)	3.5 (2.2–5.2)	2.5 (1.6–3.9)	<0.001
<1.0 µg/day (%) (*n*)	9.2 (67)	4.5 (13)	12.1 (54)	<0.001
Top food group contributors	Meat (53%), Fish (17%), Milk (13%)	Meat (59%), Fish (16%), Milk (10%)	Meat (48%), Fish (19%), Milk (15%)	-
Plasma Vitamin B12 (pmol/L)	232 (170–324)	228 (166–309)	238 (174–337)	0.238
<148 pmol/L (%) (*n*)	17.1 (125)	17.4 (50)	16.9 (75)	0.841

BMI, body mass index; Cereals, Cereals and cereal products; Fruit, Fruit and fruit juice; Meat, Meat and meat products; Fish, Fish and fish dishes; Milk, Milk and milk products; PPI, proton pump inhibitors. Values are medians and IQR unless otherwise stated. ^1^ No sex difference by Chi-squared test (χ^2^) for categorical or Mann–Whitney test for non-parametric continuous variables; ^2^ Independent *t*-test.

**Table 2:** Plasma vitamin B12 and RBC folate concentrations by *FUT2, MTHFR*, *MTR*, and *TCN1* genotypes in the Newcastle 85+ Study.

	RBC Folate (nmol/L)	*p*-Value ^1^	Plasma Vitamin B12 (pmol/L)	*p*-Value ^1^
***FUT2*** (rs492602)		0.531		<0.001
AA (*n* = 128)	894 (629–1349		216 (146–281)	Ref.
A/G (*n* = 308)	917 (603–1322)		221 (163–309)	0.413
GG (*n* = 187)	835 (595–1206)		277 (209–381)	<0.001
***MTHFR*** (rs1801133)		0.028		0.244
GG (*n* = 276)	871 (614–1275)	Ref.	234 (168–331)	
A/G (*n* = 279)	845 (584–1263)	1.000	230 (164–312)	
AA (*n* = 67)	1010 (693–1626)	0.060	249 (193–339)	
***MTR*** (rs1805087)		0.547		0.277
AA (*n* = 419)	881 (613–1278)		240 (173–337)	
A/G (*n* = 178)	845 (596–1332)		226 (162–297)	
GG (*n* = 26)	1053 (580–1593)		247 (162–310)	
***TCN1*** (rs526934)		0.065		0.298
AA (*n* = 331)	877 (606–1317)		237 (178–336)	
A/G (*n* = 247)	845 (595–1223)		231 (160–325)	
GG (*n* = 45)	1074 (630–1439)		222 (182–273)	

RBC folate, Red blood cell folate; *FUT2*, Fucosyltrasnferase 2; *MTHFR*, Methylenetetrahydrofolate reductase; *MTR*, Methionine synthase; *TCN1*, Transcobalamin 1. Ref., Reference used for post hoc comparisons. ^1^ Kruskal–Wallis test followed by Dunn–Bonferroni post-hoc test if the null hypothesis was rejected.

**Table 3:** Odds ratio (95% CI) of low RBC folate concentration according to quartiles of total folate intake and intakes from cereals and cereal products, from vegetables and from fruit and fruit juice in the Newcastle 85+ Study.

Folate Intake	Model 1 (Unadjusted)		Model 2 (Adjusted)	
Total (µg/day)	<600 nmol/L (*n* = 170)	*p*	<600 nmol/L (*n* = 170)	*p*
<157	1.00 (ref.)	-	1.00 (ref.)	-
157–208	0.64 (0.40, 1.04)	0.071	0.65 (0.38, 1.09)	0.103
209–264	0.72 (0.45, 1.15)	0.173	0.58 (0.34, 1.02)	0.057
>264	0.58 (0.36, 0.94)	0.028	0.43 (0.23, 0.82)	0.010
Cereals and Cereal products (µg/day)	<600 nmol/L (*n* = 170)	*p*	<600 nmol/L (*n* = 170)	*p*
<36	1.00 (ref.)	-	1.00 (ref.)	-
36–59	0.96 (0.61, 1.49)	0.840	0.84 (0.51, 1.38)	0.493
59–92	0.40 (0.24, 0.66)	<0.001	0.32 (0.18, 0.57)	<0.001
>92	0.41 (0.25, 0.68)	0.001	0.33 (0.18, 0.61)	<0.001
Vegetables (µg/day)	<600 nmol/L (*n* = 154)	*p*	<600 nmol/L (*n* = 154)	*p*
<15	1.00 (ref.)	-	1.00 (ref.)	-
15–30	0.72 (0.43, 1.21)	0.212	0.49 (0.25, 0.95)	0.035
30–51	0.86 (0.52, 1.41)	0.550	0.59 (0.32, 1.08)	0.089
>51	0.79 (0.48, 1.30)	0.357	0.52 (0.28, 0.99)	0.045
Fruit and Fruit Juice (µg/day)	<600 nmol/L (*n* = 127)	*p*	<600 nmol/L (*n* = 127)	*p*
<7.3	1.00 (ref.)	-	1.00 (ref.)	-
7.3–16	0.90 (0.53, 1.52)	0.682	1.01 (0.56, 1.83)	0.979
16–34	0.61 (0.35, 1.07)	0.086	0.67 (0.36, 1.25)	0.213
>34	0.76 (0.44, 1.31)	0.329	0.79 (0.43, 1.44)	0.437

RBC folate, Red blood cell folate; *p*, *p*-value. Low folate status was defined as RBC folate concentration <600 nmol/L. Binary logistic regression model. Model 1 is unadjusted and Model 2 is adjusted for sex, energy intake, folate intake from the other two food sources (except for total folate), *MTHFR* genotype and folic acid-containing supplement use.

**Table 4:** Odds ratio (95% CI) of plasma vitamin B12 deficiency according to quartiles of intake of total vitamin B12 and intakes from meat and meat products, from fish and fish products, and from milk and milk products in the Newcastle 85+ Study.

Vitamin B12 Intake	Model 1 (Unadjusted)		Model 2 (Adjusted)	
Total (µg/day)	<148 pmol/L (*n* = 125)	*p*	<148 pmol/L (*n* = 125)	*p*
<1.87	1.00 (ref.)	-	1.00 (ref.)	-
1.87–2.88	0.70 (0.42, 1.18)	0.180	0.57 (0.32, 1.01)	0.056
2.88–4.40	0.60 (0.35, 1.02)	0.057	0.50 (0.28, 0.92)	0.026
>4.40	0.53 (0.31, 0.92)	0.024	0.40 (0.21, 0.76)	0.005
Meat and Meat products (µg/day)	<148 pmol/L (*n* = 118)	*p*	<148 pmol/L (*n* = 118)	*p*
<0.35	1.00 (ref.)	-	1.00 (ref.)	-
0.35–1.03	0.72 (0.42, 1.24)	0.236	0.69 (0.38, 1.25)	0.220
1.03–2.10	0.84 (0.50, 1.44)	0.533	0.78 (0.43, 1.42)	0.422
>2.10	0.55 (0.31, 0.98)	0.043	0.41 (0.20, 0.81)	0.010
Fish and Fish products (µg/day)	<148 pmol/L (*n* = 43)	*p*	<148 pmol/L (*n* = 43)	*p*
<0.46	1.00 (ref.)	-	1.00 (ref.)	-
0.46–1.06	0.61 (0.23, 1.65)	0.331	0.66 (0.23, 1.91)	0.444
1.06–2.45	0.86 (0.34, 2.15)	0.743	0.66 (0.23, 1.86)	0.427
>2.45	1.00 (0.41, 2.42)	0.992	0.70 (0.25, 1.97)	0.503
Milk and Milk products (µg/day)	<148 pmol/L (*n* = 102)	*p*	<148 pmol/L (*n* = 102)	*p*
<0.27	1.00 (ref.)	-	1.00 (ref.)	-
0.27–0.53	0.84 (0.47, 1.52)	0.562	0.88 (0.46, 1.71)	0.711
0.53–0.88	1.12 (0.64, 1.96)	0.698	1.28 (0.70, 2.37)	0.425
>0.88	0.58 (0.31, 1.08)	0.086	0.49 (0.24, 1.01)	0.054

*p*, *p*-value. Binary logistic regression model. Deficient plasma vitamin B12 concentration was defined as <148 pmol/L. Model 1 is unadjusted and Model 2 is adjusted for sex, energy intake, *FUT2* genotype, vitamin B12 intake from the other two food sources (except total vitamin B12 intake), vitamin B12 containing supplement use, H_2_ antagonists, biguanides and proton pump inhibitors use.
